# Validity and reliability of the Sri Lankan version of the kidney disease quality of life questionnaire (KDQOL-SF™)

**DOI:** 10.1186/s12955-017-0697-6

**Published:** 2017-06-05

**Authors:** Sameera Senanayake, Nalika Gunawardena, Paba Palihawadana, Sanjeewa Kularatna, T. S. G. Peiris

**Affiliations:** 1 Epidemiology Unit, Ministry of Health, Colombo, Sri Lanka; 2 World Health Organization Country Office, Colombo, Sri Lanka; 3 Australian Centre for Health Services Innovation, Queensland University of Technology, Brisbane, Australia; 4 Department of Mathematics, University of Moratuwa, Colombo, Sri Lanka

**Keywords:** Chronic kidney disease, Quality of life, Sri Lanka, Chronic kidney disease of unknown aetiology

## Abstract

**Background:**

The disabling symptoms, various food and fluid restrictions, restrictions to social life and stigma and taboos attached to Chronic Kidney Disease (CKD), have shown to pose a significant bearing on a patient’s Quality of Life (QOL). In the present study the Kidney Disease QOL-Short Form (KDQOL-SF™) was culturally adapted, modified and translated into Sinhala and validity and reliability were assessed.

**Method:**

The process to culturally adapt the Kidney Disease Specific Component (KDSC) of KDQOL-SF™ was carried out by the modified Delphi process with a group of experts. The construct validity of the KDSC was assessed using Exploratory Factor Analysis (EFA). Appraising construct validity of SF-36 component of KDQOL-SF™ was done by assessing the convergent and discriminant validity using the Multitrait-Multimethod Matrix technique (MTMM). Randomly selected 250 CKD patients attending the five renal clinics in Polonnauwa were used to assess the construct validity. To assess the test-retest reliability of the instrument, within a period of one week, 30 randomly selected study participants were visited at their households.

**Results:**

Two hundred and fifty adults with documented evidence of CKD participated. The EFA carried out using principal component factoring method and rotated by Varimax orthogonal method resulted in 14 factors with Eigen values ranging from 1.062–8.746. This 14 factor model explained 84.1% of total variance of the initial system. The communalities extracted for domains were all close to one. All the items were loaded to one or more domains with factor coefficients of more than 0.4, not requiring any of the items to be dropped. Few items which showed similarly high factor coefficients in more than one factor were assigned to a factor ensuring the pattern in the theoretical framework of the questionnaire based on expert opinion and vigorous analysis of literature.

Convergent and divergent validity assessed using MTMM, revealed satisfactory construct validity. Cronbach’s alpha of all domains of KDQOL-SF™ except for cognitive function and Social function, exceeded Nunnally's criteria of 0.7. The Intra class Correlation Coefficients (ICC) were more than 0.8 for all the domains, which indicated good test re-test reliability.

**Conclusions:**

KDQOL-SF™ is a valid and reliable instrument which can be used to assess QOL of CKD patients in Sri Lanka.

## Background

The prevalence of Chronic Kidney Disease (CKD) is increasing globally, though regional disparities exist. It is increasing worldwide at an annual growth rate of 8% [1]. Epidemiologic studies have shown that the incidence of kidney diseases is higher in the developing countries than in the developed world.

Quality of Life (QOL) is a subjective phenomenon which can be influenced by a person’s experience, beliefs and expectations [2]. Despite enormous advances in treatment modalities, the disabling symptoms, various food and fluid restrictions, restrictions to social life and stigma and taboos attached to CKD, have shown to pose a significant bearing on a patient’s QOL [3].

Several studies demonstrated strong relationship between reduced QOL and increased morbidity and mortality related to CKD. The importance of including QOL indicators to the clinical management of CKD patients has been highlighted world over [4–7].

Various tools have been developed to quantitatively measure disease related QOL, but these tools need disease and country specific validation [8]. The Kidney Disease Quality Of Life-Short Form (KDQOL-SF™) version 1.3 questionnaire is a tool used to measure the QOL of kidney disease patients. It is a self-administered tool developed in 1995 by Hays et al., specifically to assess QOL among CKD patients. It has been translated into several languages including Dutch, Korean, Italian, Iranian, Brazilian, and Japanese and has been validated in various countries.

Evaluation of QOL among CKD patients in Sri Lanka can add new insight into the management of the disease as it allows the quantification of the disease consequences according to the patient’s perception and enables adjustment of medical decisions to their physical, emotional, and social needs [9].

In the present study the KDQOL-SF™ version 1.3 was culturally adapted, modified and translated into Sinhala, which is the most common language spoken in Sri Lanka. This Sinhala version of the KDQOL-SF™ 1.3 was subsequently tested among a representative sample of Sri Lankan CKD patients to determine the psychometric properties, reliability, and validity for use in the assessment of QOL among CKD patients.

## Methods

### Structure of KDQOL-SF™ version 1.3

KDQOL-SF has two components; Kidney Disease Specific Component and SF-36. Of the total 81 questions in 19 domains, 43 questions assess 11 kidney disease specific components of QOL and SF-36 questionnaire in which the 36 questions assess the general health related QOL in eight domains.

The 11 domains of Kidney Disease Specific Component are: symptom/problem list (12 items), effects of kidney disease (8 items), burden of kidney disease (4 items), cognitive function (3 items), quality of social interaction (3 items), sexual function (2 items), sleep (4 items), social support (2 items), work status (2 items), patient satisfaction (1 item), and dialysis staff encouragement (2 items). SF-36 includes 36 items that measure eight domains and the eight domains are: physical function (10 items), role limitations caused by physical problems (4 items), role limitations caused by emotional problems (3 items), pain (2 items), general health perceptions (5 items), social function (2 items), emotional well-being (5 items), and energy/fatigue (4 items). The final item, the overall health rate item, asks the respondents to rate their health on a 0–10 response scale. Different questions have different answer options, which range from two to seven. When scoring, each question is scored in a scale ranging from 0 (worst health) to 100 (best health). All items in a domain are summed up and averaged to give an average score for each domain which ranges from 0 (worst health) to 100 (best health).

### Cultural adaptation and modification of kidney disease specific component of KDQOL-SF™

Considering the fact that Sri Lanka is a developing country, it is necessary that some of the words and examples used in the Kidney Disease Specific Component of KDQOL-SF™ undergo cultural adaptation before it can be applied to Sri Lanka. Therefore, the Kidney Disease Specific Component of KDQOL-SF™ was subjected to cultural adaptation and modification in the present study. The culturally adapted Kidney Disease Specific Component of KDQOL-SF™ was subjected to pre-testing and assessments of content and face validity and construct validity as well as reliability.

The process to culturally adapt the Kidney Disease Specific Component of KDQOL-SF™ was carried out by the modified Delphi process [10] with a group of experts (*n* = 15) from the fields of nephrology, general medicine, psychiatry and community medicine.

The experts were requested to review each items in the questionnaire and to indicate whether the item should be retained in the questionnaire to assess the HRQOL of CKD patients in Sri Lanka. If they decided that the item should retain, then they were asked to assess the cultural appropriateness of the words and examples used in the items in a 1–5 scale. If they assign a score less than three, they were further asked to indicate their suggestion on how the item should be modified to improve the cultural appropriateness. In the first round, they were also asked to indicate any additional kidney disease specific items/areas they think are relevant to HRQOL of CKD patients in Sri Lanka

Agreement of more than 50% of the expert panel to remove an item, was taken as the cut off to remove an item from the questionnaire. An average score of four or above was taken as an agreement of the experts on cultural appropriateness of an item. If the average score of an item was less than four, the modifications suggested by the experts to improve the cultural appropriateness were reviewed and the best option was selected.

The communications were made via individually addressed electronic mails and the identity of individual panelists was not revealed to the others until the end of the process.

The results of the first round of iteration showed that more than 50% of the experts indicating removal of the item 13, “Problems with your catheter site”, and all the other items were marked as culturally appropriate, to be used to assess QOL of CKD patients in Sri Lanka.

The rating for each item and all additional remarks were summarized by the principal investigator (PI). In the first round, the mean scores for each item ranged from 3.44 to 5.00. Items 01, 20, 27, 36, 43, 44 and 45 had a mean score less than 4.0 indicating that the wordings should be modified to make it more culturally appropriate. These items were modified by reviewing the modifications suggested by the experts (Table 1).

**Table 1:** Modifications to items according to the suggestions by the expert panel

Item number	Previous item	Modified/new item
01	Soreness in your muscles	Muscle aches and pains
20	Your sex life	Having sexual relationships with your partner
27	Does your health keep you from working at a paying job	Do you have to stay away from your job due to your ill health?
36	Becoming sexually aroused	Desire for sexual activity
43	Dialysis staff encourage me to be as independent as possible	Hospital staff encourage me to be as independent as possible
44	Dialysis staff support me in coping with my kidney disease	Hospital staff support me in coping with my kidney disease
45	Think about the care you receive for kidney dialysis.	Think about the care you receive for kidney disease.

In the second round, the modified tool with the mean scores of each item in the first round was communicated to the expert panel. The expert panel was asked to rate the cultural appropriateness of the words used in the modified items in a 1–5 scale. Upon summarizing the scores of each modified item, it was shown that the mean score of all the items were above 4.0.

### Translation of kidney disease specific component of KDQOL-SF™

Two translators, both with a high level of proficiency in English and Sinhala, independently translated the questionnaire into Sinhala. The two Sinhala translated versions were then reviewed by an independent expert proficient in both English and Sinhala languages. The items which were agreed upon were accepted for forward translation. Two bilingual translators, both with a high level of proficiency in English and Sinhala, independently translated the provisional forward translation back into English without referring to the original English version. The two English translated versions were then reviewed by an independent expert, proficient in both English and Sinhala languages.

### Pretesting of the KDQOL-SF™

The SF-36 component has been previously culturally adapted, translated to Sinhalese and validated among different population groups in Sri Lanka [11]. Thus, the already validated SF-36 component of KDQOL-SF™ was pre-tested among a group of CKD patients. Further, its content and face validity and convergent-discriminant validity as well as reliability were appraised in the present study.

Pre-testing the KDQOL-SF™ among ten CKD patients was done using the services of the renal clinic at Maligawatta National Institute for Nephrology Dialysis and Transplantation in the district of Colombo. Administration of each questionnaire was followed by a structured interview conducted by the PI. Based on the interviews, a few modifications were done to the instructions stated for some items.

### Appraising construct validity and reliability of the KDQOL-SF™

A multidisciplinary panel of experts appraised and confirmed the face, content and consensual validity of the tool. As the factorial validity of the Kidney Disease Specific Component of KDQOL-SF is not established in many different settings elsewhere, it was decided to carry out Exploratory Factor Analysis (EFA) on the observations of the validation study to identify the common factors of the original system of 43 dimension. Prior to EFA, factorability of the data was assessed by Bartlett’s test of sphericity, which showed that the population correlation matrix is significantly different from the identity matrix (Bartlett Test Statistics 3907.89; *p* < 0.001). The Kaiser-Meyer-Olkin measure which is an index for comparing the magnitude of the observed correlation matrix to the magnitude of the partial correlation matrix (0.791) was greater than the recommended value for factor analysis. Also overall reliability index was assessed using Cronbach’s alpha (0.889) and it was greater than 0.8. Thus the above results confirmed that the data set can be used for factor analysis.

Appraising construct validity of SF 36 component of KDQOL-SF™ was done by assessing the convergent and discriminant validity. To assess convergent and discriminant validity of SF-36 component of KDQOL-SF™, World Health Organization QOL –BREF (WHOQOL-BREF) which is a generic tool to assess QOL and which has been validated in Sri Lanka was used [12]. Comparable domains of both tools were used for the assessment of convergent and discriminant validity. Convergent and discriminant validity were measured using the Multitrait-Multimethod Matrix technique (MTMM).

The three hospitals in Polonnaruwa district, namely; District General Hospital Polonnauwa, Base Hospital Medirigiriya and Divisional Hospital Hingurakgoda, conduct five renal clinics per week. To assess the construct validity, 50 CKD patients were randomly selected from each above said five clinics using the clinic register as the sampling frame. A total of 250 patients were thus selected. The required number of 250 study participants were distributed equally and from each clinic, 50 eligible study participants were selected using convenient sampling method. The patients who are over 18 years old and who were diagnosed as having CKD, irrespective of whether the etiology is known or unknown, by a consultant nephrologist or by a consultant physician were included in the study. The patients who had a previous renal transplantation and patients who were critically ill, from whom reliable information cannot be acquired were excluded from the study.

To assess the test-retest reliability of the study instrument, within a period of one week, 30 randomly selected study participants were visited at their households by the data collectors. Test re-test reliability of both components of KDQOL-SF™ was assessed using Intra class Correlation Coefficient (ICC) and a value of 0.70 or greater was considered as satisfactory reliability [13].

Internal consistency of both components of KDQOL-SF™ was assessed by calculating the Cronbach’s alpha. Internal consistency estimates for each of the domains of a magnitude of 0.7 or greater according to Nunnally’s criterion, was considered as satisfactory internal consistency [14].

### Quality of data

All possible measures were taken to ensure the quality of information gathered while collecting data. Data collectors were experienced in functioning as data collectors for many local and international studies done among CKD patients in the NCP and further they were trained by the PI to ensure the quality of the data collected. The study participants were informed of the purpose of the study, nature of the study and the fact that the data collected would be confidential and accessible only to the PI.

Data entry and re-checking was done by the PI. Any inconsistent or missing responses were traced back to the questionnaires and were corrected.

### Data analysis

Scoring of the two study instruments (KDQOL-SF™ and WHOQOL-BREF) were carried out according to the instructions provided by the original authors. The Statistical Package for the Social Sciences (SPSS) analytic software version 20 was used for the analysis.

## Results

### Basic information

Two hundred and fifty adults with documented evidence of CKD participated in the validation study. The mean age of the participants was 57.7 years (SD 10.6). The majority of the participants were females (*n* = 145; 58.0%). A majority of those who were currently employed in the study population, were farmers (*n* = 85; 73.3%). Details of the CKD conditions were obtained from the medical records, which showed that the mean number of years since the diagnosis of CKD was 6.3 years (SD = 3.7). The mean eGFR value was 29.3 ml/min/1.73 m2 (SD = 19.0). A majority (*n* = 179; 71.6%) were in either CKD category three or category four. Documented Chronic Kidney Disease of Unknown origin (CKDu) was seen in 20.8% (*n* = 52). For more details refer Table 2. The descriptive statistics of the KDQOL-SF™ are summarized in Table 3.

**Table 2:** Distribution of the study population by socio demographic and renal related characteristics

Socio demographic characteristics		(*N* = 250) *n*	Percent
Age categories (Years)	18–40	17	6.8
	41–60	130	52.0
	61–80	103	41.2
Sex	Male	105	42.0
	Female	145	58.0
Highest level of education	Never gone to school	24	9.6
	Primary education	93	37.2
	Junior secondary education	95	38.0
	Senior secondary education	32	12.8
	Collegiate	05	2.0
	University	01	0.4
GFR category	Stage 1	48	19.2
	Stage 2	11	4.4
	Stage 3	71	28.4
	Stage 4	108	43.2
	Stage 5 (Non dialysis)	09	3.6
	Stage 5 (Dialysis)	03	1.2

**Table 3:** Median, mean and standard deviation of the 19 domains in KDQOL SF – 36

Domains	Median (IQR)	Mean (SD)	Number of items	Internal consistency (Cronbach’s alpha)
Kidney disease targeted areas				
Symptom/problem domain	63.6 (50.0–77.3)	63.1 (18.1)	12	0.769
Effects of kidney disease	75.0 (56.2–87.5)	71.5 (19.9)	8	0.765
Burden of kidney disease	43.7 (25–62.5)	46.2 (26.7)	4	0.797
Work status	50.0 (0.0–50.0)	42.6 (30.9)	2	0.859
Cognitive function	60.0 (40.0–73.3)	57.9 (26.6)	3	0.570
Quality of social interaction	80.0 (60.0–93.3)	75.5 (19.7)	3	0.745
Sexual function	100.0 (75.0–100.0)	84.5 (22.0)	2	0.975
Sleep	62.5 (42.5–77.5)	59.3 (23.5)	4	0.708
Social support	83.3 (66.7–100.0)	78.1 (22.6)	2	0.894
Hospital staff encouragement	100.0 (100.0–100.0)	93.6 (16.0)	2	0.906
Patient satisfaction	66.6 (50.0–83.3)	58.1 (22.1)	1	Not applicable
Physical Component Summary				
Physical functioning	50.0 (15.0–75.0)	46.8 (32.1)	10	0.945
Role – physical	0.0 (0.0–0.0)	21.1 (39.5)	4	0.975
Pain	35.0 (22.5–57.5)	40.5 (27.2)	2	0.814
General health	40.0 (30.0–50.0)	41.1 (18.6)	5	0.701
Mental Component Summary				
Emotional well-being	52.0 (40.0–72.0)	56.2 (22.6)	5	0.874
Role – emotional	0.0 (0.0–100.0)	26.6 (43.4)	3	0.981
Social function	62.5 (50.0–100.0)	66.1 (29.8)	2	0.685
Energy/fatigue	35.0 (20.0–55.0)	37.7 (24.9)	4	0.725
Overall Health	50.0 (40.0–70.0)	51.2 (18.9)	1	Not applicable

### Exploratory factor analysis for the kidney disease specific component of KDQOL-SF™

The results of the EFA carried out by extracting factors using principal component factoring method identified 14 factors based on the eigenvalue greater than one criteria and those 14 factors were able to acquire 84% of the variability of the initial system. Those 14 factors were rotated using Varimax orthogonal method to make the factors more meaningful. The validity of the 14-factor model was further confirmed by the communalities of each attributes as all the communalities were close to one.

All the items were loaded to one or more domains with factor coefficients of more than 0.4, not requiring any of the items to be dropped (Table 4). However, some items did not load to the factors to which they belong in the original KDQOL SF™ (eg. 14 I, 14 J, 15B, 15G and 18C) and few items showed similarly high factor coefficients in more than one factor. Few items which showed similarly high factor coefficients in more than one factor were assigned to a factor ensuring the pattern in the theoretical framework of the questionnaire based on expert opinion and vigorous analysis of literature.

**Table 4:** Factor coefficients of individual items of kidney disease component of KDQOL SF TM mapped with the domain structure of the kidney disease component of KDQOL SF TM based on expert opinion and analysis of literature

Domain	Question	F1	F2	F3	F4	F5	F6	F7	F8	F9	F10	F11	F12	F13	F14
BKD	12A									0.528					
	12B									0.513					
	12C									0.556					
	12D									0.405					
QSI	13A			0.486											
	13C			0.520											
	13E			0.421											
CF	13B													0.878	
	13D													-0.604	
	13F													0.413	
Symptom/problem Domain	14A											0.789			
	14B								−0.503						
	14C								0.855						
	14D						0.744								
	14E						0.870								
	14F								−0.823						
	14G											0.433			
	14H											0.774			
	14I			0.629^a^											
	14 J				0.812^a^										
	14 K											0.840			
EKD	15A	0.649													
	15B			0.710^a^											
	15C	0.681													
	15D												0.489		
	15E	0.405													
	15F	0.725													
	15G		0.551^a^												
	15H												0.610		
SF	16A					0.933									
	16B					0.907									
Sleep	17		0.878												
	18A		0.904												
	18B		0.685												
	18C										0.825^a^				
SS	19A				0.805										
	19B				0.693										
WS	20														0.802
	21														0.720
PS	23										0.509				
HSE	24A							0.870							
	24B							0.879							

*BKD* Burden of kidney disease, *QSI* Quality of social interaction, *CF* Cognitive function, *EKD* Effects of kidney disease, *SF* Sexual function, *SS* Social support, *WS* Work status, *PS* Patient satisfaction, *HSE* Hospital staff encouragement

^a^Questions which did not load to an appropriate factors in accordance with the theoretical background

### Convergent and discriminant validity of SF-36 component of KDQOL-SF™

**Fig. 1:**
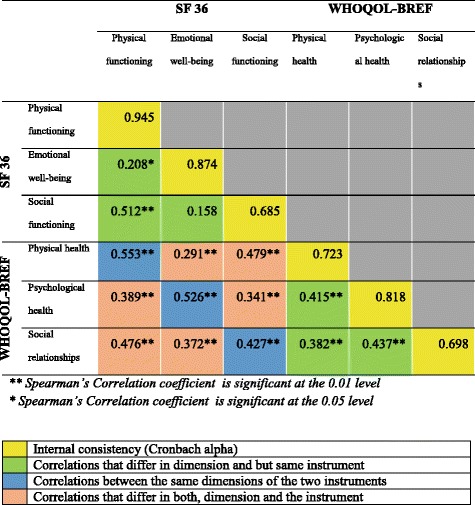
MTMM of Correlation Coefficients for SF – 36 and WHOQOL-BREF

Construct validity of the SF-36 was assessed by evaluating convergent and discriminant validity against WHOQOL – BREF. The Multitrait Multimethod Matrix (MTMM) of correlation coefficients for SF-36 component of KDQOL SF™ and WHOQOL – BREF among study population of the validation study are shown in Fig. 1.

Correlation coefficient observed between the physical functioning domain of SF - 36 and the physical functioning domain of SF-36 was 0.945. Similarly, the reliability coefficients highlighted in yellow which are also the Cronbach alphas were consistently the highest in the matrix showing that the instruments are correlated with itself than with anything else, confirming the reliability of the SF-36 component of KDQOL SF™.

Validity coefficients which are the correlations between the same dimensions of the two instruments highlighted in blue in the Fig. 1 were high except in the social functioning domain. This provided evidence for convergent validity of the SF-36 component of KDQOL SF™.

Correlations that differ in both, dimension and the instrument were the lowest in the matrix highlighted by the correlation observed between the emotional wellbeing domain of SF-36 and the physical health domain of WHOQOL-BREF among the study participants of the validation study (*r* = 0.291) being much lower than the correlation observed between the emotional wellbeing domain of SF-36 and the psychological health domain of WHOQOL-BREFF (*r* = 0.526), confirming that the SF-36 component of KDQOL SF™ is showing discriminant validity.

### Reliability of KDQOL-SF™

Cronbach’s alpha of all domains of KDQOL SF™ except for cognitive function and Social function, exceeded Nunnally's criteria of 0.7 which can be considered adequate internal consistency (Table 3). The Intra class Correlation Coefficients were more than 0.8 for all the domains indicating good test re-test reliability.

## Discussion

The KDQOL SF™ is a CKD specific study instrument. The use of specific tools to assess the QOL has been found to generate results which can be readily translated to relevant recommendations to uplift the QOL of the diseased. Such specific assessments are also more acceptable to nephrology fraternity. KDQOL SF™ is a tool that has been used widely in the world to assess HRQOL among CKD patients [8], making the results generated comparable to evaluate the differences. The mean completion time of the tool was found to be approximately 20 min which is shorter than in many other QOL instruments [15].

The present study validated the KDQOL SF for Sri Lanka and this is the first attempt in Sri Lanka to validate a QOL instrument for CKD patients. Though KDQOL SF™ has been validated and used in many countries with evidence of high validity and reliability [16], it was imperative that the tool be culturally adapted and the validity of the tool to assess QOL of the CKD patients in the Sri Lankan rural community be assessed prior to use [8].

The KDQOL SF™ comprises two clearly defined components; Kidney Disease Specific Component and SF-36. The SF 36 component of the KDQOL SF™ has previously been culturally adapted, translated and validated in different settings in Sri Lanka [11] and the present study used the validated version to improve the efficiency of the study. The cultural adaptation of the Kidney Disease Specific Component was through the guidance of a panel of experts using the Delphi technique. The service of a panel of experts from different professional backgrounds, some of whom have been directly involved in the caring of CKD patients in Anuradhapura district, enabled the cultural suitability to be assessed in different perspectives. The use of the Delphi technique instead of face to face consultative meetings had the advantage of not requiring the experts to take time off their schedules to contribute to the study. It allowed the experts to respond at any time convenient to them and to contact any source of information if needed. Further, this process facilitated the independence of forming opinion and perspectives [10].

Exploratory Factor Analysis performed using Principal Component Analysis revealed the measures of sample adequacy, Kaiser-Meyer-Okin measure to be close to one (0.791) and Bartlett’s Test for Sphericity to be statistically significant, indicating that the sample was adequate. Results of EFA on kidney disease specific component of KDQOL-SF™ revealed 14 factors with Eigen values more than one (ranging from 1.062–8.746),when factors were extracted using PCA. PCA is an accepted method of selecting factors in EFA [17]. The total variance explained by the model was 84.1% which indicate that the 14 factors identified explain the 84.1% of the total variability of 43 items in the original kidney disease specific component of KDQOL-SF™, which can be considered acceptable. The communalities extracted for domains were all high, (ranging from 0.711 to 0.951) which indicated high correlation between the items. Though within each factor, all the items loaded into one or more factors, not requiring any of the items to be dropped, cross loadings were apparent between some factors (eg; 12A, 13E, 14B) which can be explained by the overlapping nature of assessed variables.

The study adopted a process, so that the factor structure was not decided solely based on statistical results, but by an approach suggested by the experts and global literature.

Exploratory Factor Analysis performed on the Kidney Disease Specific Component has yielded different results globally. EFA performed on a modified Egypt version of KDQOL SF™, has resulted in a ten factor model explaining 70.9% of the variability [8]. When principal components analysis was performed, all 43 items explained 79.81% of the total variance in 11 factors in the Iranian version [16]. QOL is a construct which is very much related to the socio-cultural and economical background of a setting, thus these different results found in different settings could be attributed to these differences.

The Multitrait Multimethod Matrix (MTMM) of correlation coefficients, performed to assess convergent and discriminant validity of the SF-36 component of KDQOL SF™, resulted in the expected correlations for convergent and discriminant validity except in the social functioning. This can be taken as evidence of good validity of the SF-36 component of KDQOL-SF™ to assess HRQOL of CKD patients in Sri Lanka. The finding of poor correlation in the domain of social functioning was also experienced by the previous researchers. Kumarapeli [12] and Dundar et al. [18] have reports on two validation studies, between WHOQOL-BREFF and SF 36, where good correlation was found in both physical and psychological domains, but poor correlation in the social domain (0.276 and 0.393 respectively).

The internal consistency of both components of KDQOL-SF™ was assessed by calculating the Cronbach’s alpha which exceeded Nunnally's criteria of 0.7 in all domains except for cognitive function (0.570) and Social function (0.685) indicating good internal consistency [14]. Social function domain is assessed using two items in KDQOL-SF™ and cognitive function is assessed using three items and these limited number of items assess diverse aspects of respective domains. In addition, the complexity of the social functions in a rural community in Sri Lanka, could be the reasons for relatively low internal consistency of the two domains.

Low internal consistency in Social function domain of KDQOL-SF™ is also a common finding across the world in validation studies. Similarly the Singapore version had low internal consistency in Social function (0.66) but all the other domains had Cronbach’s α of more than 0.70 [19]. Cronbach’s α was 0.64 in the cognitive function domain of the Korean version but it was 0.73 in the social function domain [20]. The Egypt version had the Cronbach’s α value more than 0.7 for all the domains other than social interaction (0.23) and work status (0.28) [8] while the Cronbach’s α value was more than 0.7 in all the domains in the Iranian version [16].

The test re-test Spearman’s correlations were more than 0.7 for all the domains indicating good test re-test reliability. Similar findings were evident in Egypt version [8], Iranian version [16] and in Korean version [20].

There were some limitations to this study. First, the patients were recruited from one district only, which may limit the generalizability of the findings to Sri Lanka. Second, some of the information related to HRQOL is considered to be sensitive in nature and the fact that this information was obtained utilizing an interviewer-administered questionnaire could have led to some under-reporting in the assessment of HRQOL, though many measures were taken to minimize this issue.

## Conclusion

The results of the validation study indicated that KDQOL-SF™ is a valid and reliable instrument which can be used to assess HRQOL of CKD patients.
